# Influence of early use of sodium-glucose transport protein 2 inhibitors, glucagon-like peptide-1 receptor agonists and dipeptidyl peptidase-4 inhibitors on the legacy effect of hyperglycemia

**DOI:** 10.3389/fendo.2024.1369908

**Published:** 2024-05-13

**Authors:** Siwei Deng, Houyu Zhao, Sanbao Chai, Yexiang Sun, Peng Shen, Hongbo Lin, Siyan Zhan

**Affiliations:** ^1^ Department of Epidemiology and Biostatistics, School of Public Health, Peking University, Beijing, China; ^2^ Center for Intelligent Public Health, Institute for Artificial Intelligence, Peking University, Beijing, China; ^3^ Key Laboratory of Epidemiology of Major Diseases (Peking University), Ministry of Education, Beijing, China; ^4^ School of Medicine, Chongqing University, Chongqing, China; ^5^ Department of Endocrinology, Peking University International Hospital, Beijing, China; ^6^ Big Data Center, Yinzhou District Center for Disease Control and Prevention, Ningbo, China; ^7^ Research Center of Clinical Epidemiology, Peking University Third Hospital, Beijing, China

**Keywords:** cardiovascular diseases, novel anti-hyperglycemic agents, population-based cohort, type 2 diabetes mellitus, legacy effect

## Abstract

**Background:**

A phenomenon known as legacy effect was observed that poor glycemic control at early stage of patients with newly-diagnosed type 2 diabetes (T2D) increases the risk of subsequent cardiovascular diseases (CVD). Early use of some novel anti-hyperglycemic agents, such as sodium-glucose transport protein 2 inhibitors (SGLT-2i), may attenuate this effect, but the evidence is limited.

**Methods:**

Two retrospective cohorts of newly diagnosed T2D patients from 2010–2023 were assembled using the Yinzhou Regional Health Care Database (YRHCD) with different definitions of the early exposure period - the 1-year exposure cohort and 2-year exposure cohort, which were comprised of subjects who had HbA1c measurement data within 1 year and 2 years after their T2D diagnosis, respectively. Using Cox proportional hazards models, we examined the association between high HbA1c level (HbA1c>7%) during the early exposure period and the risk of subsequent CVD. This analysis was performed in the overall cohort and three subpopulations with different treatments during the early exposure period, including patients initiating SGLT-2i or glucagon-like peptide-1 receptor agonists (GLP-1RA), patients using dipeptidyl peptidase-4 inhibitors (DPP-4i), and patients without using SGLT-2i, GLP-1RA, and DPP-4i. Besides, subgroup analyses were performed by stratifying patients into age <55 and ≥55 years.

**Results:**

A total of 21,477 and 22,493 patients with newly diagnosed T2D were included in the two final cohorts. Compared with patients with mean HbA1c ≤ 7% during the early exposure period, those with HbA1c>7% had higher risks of incident CVD, with a HR of 1.165 (95%CI, 1.056–1.285) and 1.143 (95%CI, 1.044–1.252) in 1-year and 2-year exposure period cohort. Compared to non-users, in patients initiating SGLT-2i/GLP-1RA within 1 or 2 years after T2D diagnosis, higher HbA1c level at baseline was not associated with CVD in both two cohorts. In subgroup analyses, results were generally consistent with the main analysis.

**Conclusions:**

Poor glycemic control in the early stage of T2D increased later CVD risk in Chinese adults with newly diagnosed T2D. Compared to non-users, this association was smaller and non-significant in patients receiving SGLT-2i/GLP-1RA during the early stage of T2D, indicating early use of these drugs may have the potential to mitigate legacy effects of hyperglycemia.

## 1 Introduction

Poor glycemic control is an important risk factor for increased cardiovascular events in patients with type 2 diabetes (T2D) (1–3). Originally, the legacy effect refers to a phenomenon where an early and good glycemic control can yield long-lasting benefits in reducing patients’ risk of cardiovascular disease (CVD) even after the intensive therapy is discontinued (2, 4, 5). For example, the results of the United Kingdom Prospective Diabetes Study showed that in newly diagnosed T2D patients, compared to patients receiving conventional dietary therapy, patients receiving intensive glucose therapy at early stage had a lower risk of myocardial infarction and microvascular diseases for 10 years even after the intensive treatment is discontinued (6). Conversely, in other studies, researchers also found that a phenomenon known as legacy effect was observed that an early and good glycemic control can yield long-lasting benefits in reducing patients’ risk of CVD even after the intensive therapy is discontinued, while poor glycemic control at early stage similarly exhibits a legacy effect in increasing the risk of subsequent CVD (7, 8). A study in the United States found that among newly diagnosed T2D patients, compared to patients with HbA1c levels < 6.5% within one year of T2D diagnosis, patients with HbA1c levels ≥ 6.5% were associated with increased microvascular and macrovascular events over the subsequent 10 years (8). Regarding this phenomenon, Ceriello et al. recently reported that sodium-glucose transport protein 2 inhibitors (SGLT-2i), a novel class of anti-hyperglycemic agents that significantly reduce cardiovascular risk (9–11), can attenuate the legacy effect of poor glycemic control when used early in patients with T2D (12). According to their results, when using SGLT-2i in the first two years after diagnosis, the association between a high level of HbA1c in early stage of T2D and an increased subsequent risk of CVDs was no longer evident. However, there are limited studies so far that have reported the influence of early SGLT-2i introduction on the legacy effect of poor early glycemic control. Additionally, these studies did not conduct further analyses in different subpopulations. Therefore, the evidence supporting this finding is currently insufficient. Furthermore, it remains unclear whether other novel class of anti-hyperglycemic agents except SGLT-2i, such as glucagon-like peptide-1 receptor agonists (GLP-1RA) and dipeptidyl peptidase-4 inhibitors (DPP-4i), can have similar effects.

In recent years, the prevalence of T2D as well as the incidence of CVD in diabetic populations have been rising rapidly in China (13–15). Meanwhile, the utilization of novel anti-hyperglycemic agents such as SGLT-2i, GLP-1RA, and DPP-4i has also rapidly increased among Chinese diabetic patients (16, 17). These novel anti-hyperglycemic agents possess multiple pleiotropic effects, particularly, for SGLT-2i and GLP-1RA. Previous studies have reported that in addition to controlling blood glucose levels, these agents also have significant beneficial impacts on cardiovascular protection, renal protection, and improvement of cognitive function (18–20). However, no studies in China have reported the influence of early treatment with these novel anti-hyperglycemic agents on the legacy effect of poor early glycemic control. In this background, we conducted a retrospective population-based cohort study to investigate the influence of early use of SGLT-2i, GLP-1RA and DPP-4i on the legacy effect of hyperglycemia.

## 2 Methods

### 2.1 Study design and population

We conducted a retrospective cohort study of newly diagnosed diabetic patients from 2010–2023 using the Yinzhou Regional Health Care Database (YRHCD). Yinzhou is a district of Ningbo in Zhejiang province of China. In 2020, Yinzhou had a total population of 1.6 million people, including 1.4 million permanent residents (21). The YRHCD is a data platform integrating health archives, electronic medical records (EMR), chronic disease registry, death registry, and other healthcare service information, covering over 95% of the resident population in Yinzhou district. Diabetic patients were registered in the chronic disease registry and followed up quarterly by community physicians to measure and record common health indicators including fasting plasma glucose (FPG) and glycated hemoglobin (HbA1c) (22–24). The study linked data from chronic disease registry and EMR to identify patients with T2D, if they 1) registered as T2D in the chronic disease registry; or 2) had two or more diagnoses of T2D in EMR without any diagnosis of type 1 diabetes. The newly diagnosed diabetic patients was defined by the first diagnosis of T2D with no T2D diagnosis in a 180-day washout period. Information on use of specific classes of drugs (glucose lowering, lipid lowering and antihypertensive agents), levels of blood glucose, and presence of medical conditions were extracted and linked from drug prescription, laboratory examination, and outpatient and inpatient visits, respectively. Details of data used in this study and their relationship have been reported previously (22, 25).

**Figure 1:**
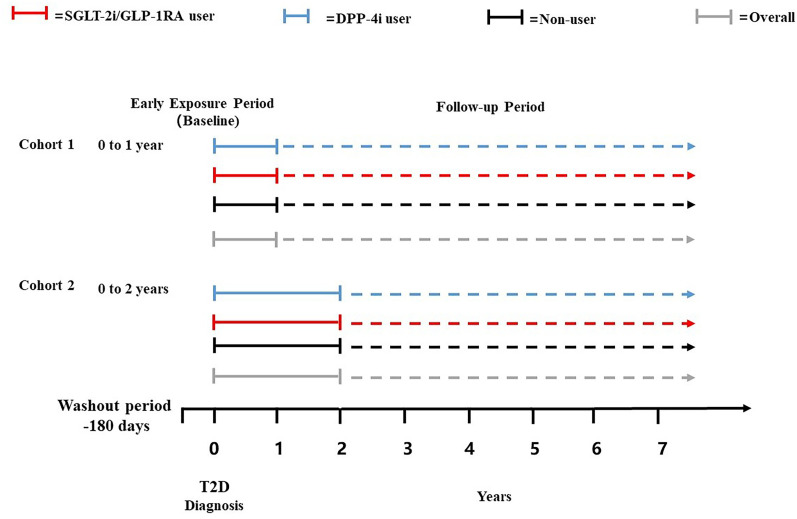
Schematic representation of the study design.

Based on prior study and the available sample size, early exposure of novel anti-hyperglycemic was defined in two ways: within one and two years after T2D diagnosis (12). The early exposure period started from the date of T2D diagnosis and ended at 1 or 2 years after diagnosis. Based on different definitions of the early exposure period, two retrospective cohorts were established - the 1-year exposure cohort and 2-year exposure cohort, which were comprised of subjects who had HbA1c measurement data within 1 year and 2 years after their T2D diagnosis, respectively. The mean HbA1c value in the period from the first day after T2D diagnosis to the last day of the exposure period was used to reflect patients’ glycemic level at baseline. HbA1c levels were categorized into two groups reflecting different degrees of glycemic control: ≤7% (≤53 mmol/mol) and >7% (>53 mmol/mol) (26). Exposure could occur at any time during the early exposure period. Due to the limited number of drug prescriptions of GLP-1RA (n=228, percentage=1.06% in 1-year exposure period cohort, with 87 patients treated by the combination of SGLT-2i and GLP-1RA; n=181, percentage=0.80% in 2-year exposure period cohort, with 70 patients treated by the combination of SGLT-2i and GLP-1RA), and the similar CVD benefits of GLP-1RA and SGLT-2i (27–29), patients who were prescribed SGLT-2i or GLP-1RA at least once during the early exposure period were classified as SGLT-2i/GLP-1RA users. Patients prescribed DPP-4i at least once were classified as DPP-4i users, and those never prescribed SGLT-2i, GLP-1RA, or DPP-4i were non-users. Patients prescribed both SGLT-2i/GLP-1RA and DPP4i during the early exposure period were excluded. Prescriptions of SGLT-2i, GLP-1RA and DPP-4i were identified using Anatomical Therapeutic Chemical (ATC) system codes as shown in the appendix (**Supplementary Table S1**). The entire follow-up period started from the first day after the end of the early exposure period, with the endpoint defined as the first occurrence of outcome, death, or censored at last visit (**Figure 1**).

The outcome was a composite of newly diagnosed myocardial infarction, heart failure, and stroke, identified by ICD-10 codes I21, I22, I50, I63-I66. Patients with a prior history of the outcome at baseline were excluded. Patients aged under 18 years were excluded from the cohort. We also excluded patients without HbA1c measured during the early exposure period, or whose endpoints occurred during the early exposure period.

### 2.2 Statistical analysis

Descriptive analyses were conducted separately for the two study cohorts. For characteristics, missing values were treated as a separate category and continuous variables were reported with means and standard deviations (SD), while categorical variables were reported with frequencies and percentages. Differences in continuous and categorical variables between groups were compared using analysis of variances and chi-square tests, respectively.

For the different treat groups in the two cohorts, Cox proportional hazards models were used to examine the association (HR) between glycemic control level during the early exposure period and risk of CVD. Age; sex; year of newly T2D diagnosed; smoking status; drinking behavior; education level; Charlson comorbidity index (CCI) (30), which was calculated according to 12 kinds of disease (**Supplementary Table S2**); use of ACEIs, ARBs, statins, calcium channel blockers (CCB), beta blockers, diuretics, other antihypertensives, other lipid lowering agents, and different classes of anti-hyperglycemic agents were included in the Cox models as covariates to adjust for confounding.

In addition to the main analysis, subgroup analyses were performed by stratifying patients into age <55 years and ≥55 years, to explore the influence of early use of different novel anti-hyperglycemic agents on the legacy effect of high level of HbA1c in early stage of T2D in different age groups.

Several sensitivity analyses were conducted to evaluate the robustness of the results. First, to avoid reverse causality, a 30-day lag time after the end of the early exposure period was set. The outcome events occurred within the 30-day lag time were excluded, and the follow-up period started 30 days after the end of the early exposure period. Second, to ensure inclusion of newly diagnosed diabetic patients, the washout period was extended to 365 days, defining newly diagnosed diabetic patients as the first diagnosis of T2D with no T2D diagnosis in a 365-day washout period. Third, given the long study period, changes in diagnostic criteria for outcomes and measurements of some covariates might have occurred over time. To avoid the potential bias, the start of study period was changed to 2018, the year after SGLT-2i were approved in China, including only patients with newly diagnosed T2D between 2018–2023. Fourth, for categorical covariates with missing values, multiple imputation was used to handle missing values. Fifth, to examine the impact of the competing risk of death on the outcomes, Fine-Gray subdistribution hazard model was used to analyze the results after adjusting for the competing risk of death. Sixth, the study classified patients who prescribed SGLT-2i at least once during the early exposure period as SGLT-2i users, and analyzed the outcomes among SGLT-2i users, DPP-4i users, and non-users (patients who never prescribed SGLT-2i or DPP-4i during the early exposure period), with GLP-1RA use included as a covariate. Seventh, to investigate the impact of different cardiovascular event definitions on the results, cardiovascular death was also included as an outcome event of interest. Finally, to further adjust for the differences in baseline covariates, inverse probability weighting (IPW) was used to correct the results.

A two-sided *p*<0.05 was considered statistically significant. Analyses were performed using SAS 9.4 (SAS Institute Inc., Cary, NC, USA).

The study was approved by the ethical review committee of the Peking University Health Science Center (IRB. No: IRB00001052–18013-Exempt). Informed consent was not required owing to the use of anonymized routine data.

## 3 Results

**Figure 2:**
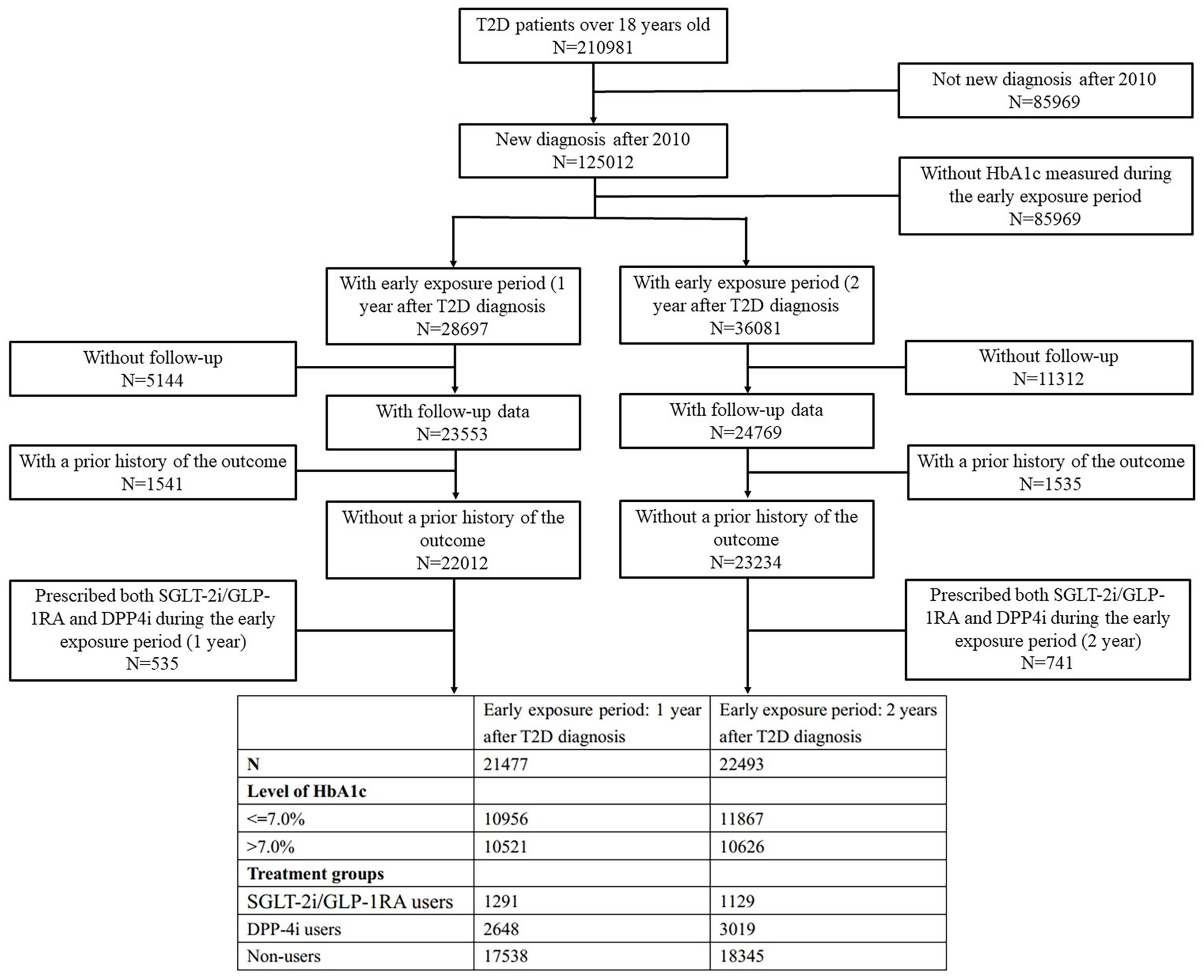
Flow-diagram showing included and excluded patients.

The two final cohorts included 21,477 and 22,493 patients with newly diagnosed T2D from 2010–2023 in YRHCD (**Figure 2**).

Based on the mean HbA1c levels during the early exposure period, each cohort was divided into HbA1c ≤ 7.0% and HbA1c>7.0% groups. In both cohorts, patients with different mean HbA1c levels differed significantly in factors including sex, smoking status, drinking behavior, education level, comorbidities, and use of antihypertensives, lipid lowering agents, and anti-hyperglycemic agents. Compared to patients with HbA1c ≤ 7.0%, patients with HbA1c>7.0% had a higher proportion of males, more likely to have smoked, and with lower education levels. In addition, patients in HbA1c>7.0% groups were also more likely to use of all kinds of anti-hyperglycemic medications (**Table 1**).

**Table 1:** Characteristics of the study population by the mean HbA1c levels during the early exposure period in two study cohort.

Variables	Early exposure period: 1 year after T2D diagnosis			Early exposure period: 2 years after T2D diagnosis		
	≤7.0%	>7.0%	*p*-value	≤7.0%	>7.0%	*p*-value
No. of patients	10956	10521		11867	10626	
Age	53.71 ± 13.05	53.58 ± 12.92	0.4945	54.22 ± 12.46	53.69 ± 12.58	0.0016
Sex (% males)	5802 (52.96)	6728 (63.95)	<0.0001	6323 (53.28)	6697 (63.02)	<0.0001
Follow-up (mean ± SD, years)	3.53 ± 2.98	3.56 ± 2.91	0.4115	3.55 ± 2.83	3.59 ± 2.85	0.3056
Smoking	2073 (18.92)	2604 (24.75)	<0.0001	2510 (21.15)	2877 (27.08)	<0.0001
Smoking, NA	2444 (22.31)	2586 (24.58)		2207 (18.6)	2126 (20.01)	
Drinking	3524 (32.17)	3363 (31.96)	0.0002	4163 (35.08)	3685 (34.68)	<0.0001
Drinking, NA	2432 (22.20)	2576 (24.48)		2200 (18.54)	2116 (19.91)	
Education						
University	1168 (10.66)	723 (6.87)	<0.0001	1118 (9.42)	710 (6.68)	<0.0001
High school	1158 (10.57)	1094 (10.40)		1228 (10.35)	1128 (10.62)	
Middle school	2757 (25.16)	2824 (26.84)		3151 (26.55)	2949 (27.75)	
Primary school	2173 (19.83)	2246 (21.35)		2627 (22.14)	2449 (23.05)	
Illiteracy	472 (4.31)	538 (5.11)		584 (4.92)	611 (5.75)	
Other	1423 (12.99)	1329 (12.63)		1576 (13.28)	1354 (12.74)	
NA	1805 (16.47)	1767 (16.79)		1583 (13.34)	1425 (13.41)	
CCI						
0	4272 (38.99)	4373 (41.56)	0.0005	4139 (34.88)	4142 (38.98)	<0.0001
1	2918 (26.63)	2756 (26.20)		3331 (28.07)	2801 (26.36)	
=2	1914 (17.47)	1771 (16.83)		2177 (18.34)	1835 (17.27)	
>2	1852 (16.90)	1621 (15.41)		2220 (18.71)	1848 (17.39)	
ACEI	1158 (10.57)	994 (9.45)	0.0062	1500 (12.64)	1175 (11.06)	0.0003
ARB	4385 (40.02)	3830 (36.40)	<0.0001	5322 (44.85)	4408 (41.48)	<0.0001
Statins	4366 (39.85)	4040 (38.40)	0.0294	5164 (43.52)	4359 (41.02)	0.0002
CCB	4160 (37.97)	3631 (34.51)	<0.0001	5106 (43.03)	4151 (39.06)	<0.0001
Beta-blocker	2246 (20.50)	1685 (16.02)	<0.0001	2700 (22.75)	1865 (17.55)	<0.0001
Diuretic	1625 (14.83)	1362 (12.95)	<0.0001	2010 (16.94)	1615 (15.2)	0.0004
Other antihypertensive agents	599 (5.47)	520 (4.94)	0.0836	654 (5.51)	558 (5.25)	0.3889
Other lipid lowering agents	1853 (16.91)	1672 (15.89)	0.0434	2173 (18.31)	1825 (17.17)	0.0260
SGLT-2i/GLP-1RA	604 (5.51)	687 (6.53)	0.0017	526 (4.43)	603 (5.67)	<0.0001
DPP-4i	1096 (10.00)	1552 (14.75)	<0.0001	1381 (11.64)	1638 (15.42)	<0.0001
Metformin	4105 (37.47)	5662 (53.82)	<0.0001	5235 (44.11)	6484 (61.02)	<0.0001
Sulfonylureas	2409 (21.99)	4164 (39.58)	<0.0001	3609 (30.41)	5197 (48.91)	<0.0001
Insulin	1016 (9.27)	2402 (22.83)	<0.0001	1312 (11.06)	2604 (24.51)	<0.0001
Alpha-glucosidase inhibitors	2099 (19.16)	3442 (32.72)	<0.0001	2854 (24.05)	3935 (37.03)	<0.0001
Glinides	616 (5.62)	942 (8.95)	<0.0001	952 (8.02)	1253 (11.79)	<0.0001
Thiazolidinediones	468 (4.27)	786 (7.47)	<0.0001	723 (6.09)	1105 (10.4)	<0.0001

T2D, type 2 diabetes; SD, standard deviations; NA, not available; CCI, Charlson comorbidity index; ACEI, angiotensin converting enzyme inhibitors; ARB, angiotensin receptor blocker; CCB, calcium channel blocker; SGLT-2i, sodium-glucose transport protein 2 inhibitors; GLP-1RA, glucagon-like peptide-1 receptor agonists; DPP-4i, dipeptidyl peptidase-4 inhibitors;

After adjusting for baseline covariates in Cox regression, results showed that compared to patients with mean HbA1c ≤ 7.0% during 1-year early exposure period, those with HbA1c>7.0% had a higher risk of incident CVD, with a HR of 1.165 (1.056–1.285). A similar pattern was observed in the cohort with a 2-year early exposure period, where the HR of patients with HbA1c>7.0% was 1.143 (1.044–1.252) (**Table 2**).

**Table 2:** Association between the mean HbA1c levels during the early exposure period and incidence of CVDs.

	Early exposure period: 1 year after T2D diagnosis		Early exposure period: 2 years after T2D diagnosis	
	HR (95%CI)	*P* value	HR (95%CI)	*P* value
Primary analysis				
Overall	1.165 (1.056–1.285)	0.002	1.143 (1.044–1.252)	0.004
SGLT-2i/GLP-1RA users	1.017 (0.520–1.989)	0.529	1.051 (0.539–2.050)	0.733
DPP-4i users	1.535 (1.026–2.299)		1.311 (0.934–1.841)	
Non-users	1.152 (1.039–1.277)		1.132 (1.028–1.246)	
Age group				
<55 years old				
Overall	1.280 (1.062–1.542)	0.010	1.199 (1.012–1.422)	0.036
SGLT-2i/GLP-1RA users	0.547 (0.137–2.179)	0.010	0.704 (0.174–2.845)	0.346
DPP-4i users	2.651 (1.236–5.686)		1.538 (0.847–2.790)	
Non-users	1.228 (1.008–1.497)		1.185 (0.989–1.421)	
≥55 years old				
Overall	1.118 (0.995–1.256)	0.060	1.114 (1.000–1.241)	0.050
SGLT-2i/GLP-1RA users	1.470 (0.625–3.457)	0.892	0.960 (0.397–2.320)	0.986
DPP-4i users	1.104 (0.669–1.822)		1.223 (0.795–1.879)	
Non-users	1.120 (0.993–1.265)		1.106 (0.988–1.240)	

T2D, type 2 diabetes; HR, hazard ratio; CI, confidence interval; SGLT-2i, sodium-glucose transport protein 2 inhibitors; GLP-1RA, glucagon-like peptide-1 receptor agonists; DPP-4i, dipeptidyl peptidase-4 inhibitors;

P values for treatment by subgroup interaction were obtained from tests of homogeneity of treatment group differences among subgroups.

Each cohort was further divided into SGLT-2i/GLP-1RA users, DPP-4i users, and non-users based on treatments during the early exposure period. The three treatment groups differed significantly in mean age, proportion of males, smoking status, drinking behavior, education level, comorbidities, and most concomitant medications. Compared with non-users, SGLT-2i/GLP-1RA users and DPP-4i users had more male patients and more patients with poor early glycemic control. Besides, SGLT-2i/GLP-1RA users and DPP-4i users also had a higher proportion of patients with CCI≥2, and more users of statins, metformin, and insulin. Detailed characteristics of the study population by treatment groups are shown in the appendix (**Supplementary Table S3**).

During the follow-up, there were 1,802 and 2,090 CVD events occurred in 1-year and 2-year exposure period cohort, respectively. The results of multivariate Cox regression analyses showed that in non-users group high level of HbA1c in early stage of T2D was significantly associated with incident CVDs with an HR of 1.152 (95%CI, 1.039-1.277) and 1.132 (95%CI, 1.028–1.246) compared with HbA1c controlled under 7.0% in 1-year and 2-year exposure period cohort, respectively. However, in patients initiating SGLT-2i/GLP-1RA within 1 or 2 years after T2D diagnosis, higher HbA1c level at baseline was not associated with CVD and the HRs were 1.017 (95%CI, 0.520–1.989) and 1.051 (95%CI, 0.539–2.050) respectively. In contrast, among DPP-4i users, patients with higher HbA1c level at baseline had higher risk of CVD and the HRs (1.535 (95%CI, 1.026–2.299) for 1-year and 1.311 (95%CI, 0.934–1.841) for 2-year exposure period cohort) were higher than that in non-users. The incidence of outcomes in different treatment groups by the mean HbA1c levels during the early exposure period in two study cohort are shown in the appendix (**Supplementary Table S4**).

In subgroup analyses, results were generally consistent with the main analysis. For example, the legacy effect of mean HbA1c>7.0% in early stage of T2D was still able to be observed in patients <55 years old with an HR of 1.280 (95%CI, 1.062–1.542) for 1-year and 1.199 (95%CI, 1.012–1.422) for 2-year exposure period cohort. Moreover, in this subgroup, compared with non-users, the association between high level of HbA1c in the early stage of T2D and increased risk of later incident CVD was still lower and non-significant in SGLT-2i/GLP-1RA users, the HRs for 1-year and 2-year exposure period cohort were 0.547 (95%CI, 0.137–2.179) and 0.704 (95%CI, 0.174–2.845), respectively (**Table 2**).

In addition, the results of all sensitivity analyses were consistent with the main analysis (**Table 3**). First, after excluding outcome events occurred within the 30-day lag time to avoid reverse causality, SGLT-2i/GLP-1RA users still showed lower and non-significant associations compared to non-users, with an HR of 1.017 (95%CI, 0.520–1.989) respectively in the 1-year exposure period cohort, and an HR of 1.031 (95%CI, 0.525–2.022) in the 2-year exposure period cohort. Second, extending the washout period to 365 days also did not change the results in the primary analysis, with HRs of 0.993 (95%CI, 0.508–1.940) and 1.011 (95%CI, 0.515–1.988) in SGLT-2i/GLP-1RA users in the two cohorts respectively. Third, when subjects were restricted to patients newly diagnosed with T2D between 2018–2023, an HR of 0.949 (95%CI, 0.478–1.882) and 0.941 (95%CI, 0.455–1.946) was observed in SGLT-2i/GLP-1RA users of two cohorts respectively. Fourth, although subjects with missing data differed significantly from those without missing data in terms of age, follow-up duration, comorbidities, and use of antihypertensives, lipid lowering agents, and anti-hyperglycemic agents (**Supplementary Table S5**), the results after multiple imputation remained consistent with the primary analysis, with the HRs of 1.064 (95%CI, 0.549–2.063) and 1.028 (95%CI, 0.529–1.996) respectively in patients initiating SGLT-2i/GLP-1RA within 1 and 2 years after T2D diagnosis. Fifth, after adjusting competing risk of death using Fine-Gray subdistribution hazard model, the HRs for SGLT-2i/GLP-1RA users in 1-year and 2-year exposure period cohort were 1.042 (0.551–1.970) and 1.048 (0.576–1.907), respectively, which were consistent with the primary analysis. Sixth, when only patients who had been prescribed SGLT-2i at least once during the early period were classified as SGLT-2i users, and GLP-1RA use was included as a covariate, the results were consistent with the primary analysis. The association between high level of HbA1c in the early stage of T2D and increased risk of later incident CVD was still lower and non-significant in SGLT-2i users, with HRs of 0.929 (0.463–1.864), and 1.013 (0.508–2.018) in the two cohorts respectively. Seventh, after including cardiovascular death as an outcome event of interest, the results remained unchanged compared to the primary analysis, with HRs of 1.017 (0.520–1.989) and 1.051 (0.539–2.050) for SGLT-2i/GLP-1RA users in 1-year and 2-year exposure period cohort, respectively. Finally, after applying IPW to adjust for the covariate differences, the results remained similar to the primary analysis. The HRs for SGLT-2i/GLP-1RA users were 1.141 (0.613–2.123) and 1.243 (0.649–2.378) in 1-year and 2-year exposure period cohort, respectively (**Table 3**).

**Table 3:** Sensitivity analyses for the association between the mean HbA1c levels during the early exposure period and incidence of CVDs.

	Early exposure period: 1 year after T2D diagnosis		Early exposure period: 2 years after T2D diagnosis	
	HR (95%CI)	*P* value	HR (95%CI)	*P* value
Sensitivity analysis: 30-day lag time				
SGLT-2i/GLP-1RA users	1.017 (0.520–1.989)	0.563	1.031 (0.525–2.022)	0.682
DPP-4i users	1.518 (1.013–2.275)		1.347 (0.951–1.908)	
Non-users	1.154 (1.039–1.281)		1.142 (1.037–1.258)	
Sensitivity analysis: 365 days washout period				
SGLT-2i/GLP-1RA users	0.993 (0.508–1.940)	0.547	1.011 (0.515–1.988)	0.657
DPP-4i users	1.513 (1.009–2.267)		1.322 (0.937–1.865)	
Non-users	1.176 (1.058–1.307)		1.132 (1.026–1.249)	
Sensitivity analysis: Study started from 2018				
SGLT-2i/GLP-1RA users	0.949 (0.478–1.882)	0.485	0.941 (0.455–1.946)	0.739
DPP-4i users	1.400 (0.882–2.222)		1.221 (0.766–1.947)	
Non-users	1.128 (0.933–1.363)		1.323 (1.053–1.662)	
Sensitivity analysis: Dataset after multiple imputed				
SGLT-2i/GLP-1RA users	1.064 (0.549–2.063)	0.541	1.028 (0.529–1.996)	0.759
DPP-4i users	1.525 (1.019–2.282)		1.316 (0.937–1.848)	
Non-users	1.153 (1.040–1.278)		1.133 (1.029–1.247)	
Sensitivity analysis: Subdistribution hazard function				
SGLT-2i/GLP-1RA users	1.042 (0.551–1.970)	0.550	1.048 (0.576–1.907)	0.751
DPP-4i users	1.529 (1.036–2.256)		1.307 (0.925–1.845)	
Non-users	1.147 (1.035–1.271)		1.132 (1.028–1.246)	
Sensitivity analysis: GLP-1RA use included as a covariate				
SGLT-2i users	0.929 (0.463–1.864)	0.462	1.013 (0.508–2.018)	0.663
DPP-4i users	1.599 (1.068–2.396)		1.307 (0.932–1.833)	
Non-users	1.153 (1.040–1.278)		1.134 (1.031–1.248)	
Sensitivity analysis: Adjusting with IPW				
SGLT-2i users	1.141 (0.613–2.123)	0.305	1.243 (0.649–2.378)	0.643
DPP-4i users	1.587 (1.073–2.348)		1.422 (1.030–1.965)	
Non-users	1.155 (1.048–1.274)		1.154 (1.054–1.264)	
Sensitivity analysis: Cardiovascular death was included in the outcome				
SGLT-2i users	1.017 (0.520–1.989)	0.520	1.051 (0.539–2.050)	0.684
DPP-4i users	1.592 (1.061–2.388)		1.320 (0.941–1.853)	
Non-users	1.145 (1.034–1.267)		1.123 (1.022–1.235)	

T2D, type 2 diabetes; HR, hazard ratio; CI, confidence interval; SGLT-2i, sodium-glucose transport protein 2 inhibitors; GLP-1RA, glucagon-like peptide-1 receptor agonists; DPP-4i, dipeptidyl peptidase-4 inhibitors; IPW, Inverse Probability Weighting;

P values for treatment by subgroup interaction were obtained from tests of homogeneity of treatment group differences among subgroups.

## 4 Discussion

To our knowledge, this study is the first to investigate the potential effects of early use of SGLT-2i/GLP-1RA and DPP-4i on remedying the legacy effect of high level HbA1c in a Chinese population with newly diagnosed diabetes. In our study, based on different definitions of the early exposure period, two retrospective cohorts were established. The 1-year exposure cohort was comprised of subjects who had HbA1c measurement data within 1 year of their T2D diagnosis, while for the 2-year exposure cohort, subjects with HbA1c measurement data within 2 year after their T2D diagnosis were included. We found that poor glycemic control within 1 or 2 years after diabetes diagnosis did increase the risk of subsequent CVD. Moreover, compared to that in non-users of SGLT-2i, GLP-1RA and DPP-4i, in patients initiating SGLT-2i/GLP-1RA, high level of HbA1c in early stage of diabetes might not increase CVD risk compared lower level of HbA1c, indicating that these drugs might have the potential to mitigate or even eliminate the harmful CVD effects of high poor glycemic control within 1 or 2 years after diabetes. In contrast, DPP-4i did not present this effect. These results were consistent in subgroup analyses and across all sensitivity analyses.

Our findings were supported by previous studies. The legacy effect of high level of HbA1c in early stage of T2D on later CVD risk that we observed in both cohorts has been reported in several prior observational studies using real-world data. An observational study from U.S. found that HbA1c levels ≥6.5% during the early 0–1 years after diabetes diagnosis were associated with increased subsequent microvascular and macrovascular events (8). Another study based on population from the United Kingdom showed that patients with HbA1c consistently above 7% within 1 year of diabetes diagnosis had significantly higher CVD rates compared to those with HbA1c <7% (7). Additionally, in the study by Ceriello et al., researchers observed a significant trend toward an increasing risk of subsequent CVDs with progressively higher levels of mean HbA1c in the early 0–1, 0–2, and 0–3 years after diabetes diagnosis (12). Ceriello et al. also found that compared to non-users, patients using SGLT-2i during the early exposure period had a lower HR for the association between high level of HbA1c in early stage of T2D and increased later CVD risk, which was also consistent with our findings (12).

Relevant mechanistic studies indicate the legacy effect may be mediated through pathways including epigenetic modifications, non-enzymatic glycosylation of proteins and chronic inflammation, and endothelial dysfunction induced by hyperglycemia-related oxidative stress (5, 31, 32). Recent researches shows that SGLT-2i and GLP-1RA, which can provide cardiovascular benefits, may have the potential to mitigate these harmful legacy effects through their mechanisms of action. Multiple studies indicate that SGLT-2i can reduce oxidative and endoplasmic reticulum stress (33–37), while GLP-1RA can repair cardiovascular damage from hyperglycemia by modulating inflammatory responses and reversing endothelial dysfunction (38–40). These findings from mechanistic studies also supported the phenomena observed in our study.

In our study, we also observed an unexpected finding that DPP-4i use during the early exposure period after diabetes diagnosis did not present the potential to mitigate the harmful legacy effects of high HbA1c level. We thought the following factor may have contributed to this phenomenon. According to the current evidence, compared to SGLT-2i and GLP-1RA, DPP-4i has smaller and less definitive effects on reducing cardiovascular risk. A network meta-analysis of 23 clinical trials found that DPP-4i did not reduce the risk of any CVDs versus placebo, and was associated with higher cardiovascular risk compared to SGLT-2i and GLP-1RA (28). Similarly, multiple observational studies have also found that patients using SGLT-2i and GLP-1RA had lower risk of CVD compared to those using DPP-4i (41–43). However, our study was only an observational study, the potential of DPP-4i inhibitors in mitigating the harmful legacy effects of high HbA1c levels still requires further analysis through future mechanistic-based pharmacological or molecular research studies.

Our study investigated and compared the legacy effect of high level of HbA1c in early stage of T2D on later CVDs between different treatment groups using real-world data from China. In the sensitivity analyses, we considered the potential impact of time-related biases like reverse causality, the potential misclassification from shorter washout periods, the potential changes in diagnostic criteria for outcomes and measurements of some covariates over time, the potential influence of missing values in some categorical covariates, as well as the potential impact of competing risk of death and differences in baseline covariates. This ultimately verified the robustness of the results. Our results validated previous findings and also provided novel evidence on DPP-4i users and across age groups. However, there were also some limitations in our study. First, due to the late market approval of SGLT-2i and GLP-1RA in China, sample sizes of users of these drugs were small, so we could not determine if the non-significant interaction between treatment and glycemic level was due to insufficient power. The limited sample size also precluded analyses in more subgroups to explore results in different subpopulations. This limitation may also impact the generalizability and robustness of our findings, and can be addressed by updating the study results after more medication users have accumulated in the database over time. Second, the follow-up duration of this study was relatively short, and the treatment group had an even shorter follow-up duration due to the late introduction of SGLT-2i and GLP-1RA in China, so we could not observe long-term effects of SGLT-2i/GLP-1RA on CVD risk. Third, residual confounding from unmeasured factors like disease severity cannot be excluded. Fourth, based on the study design, we did not include patients with prevalent CVD, nor did we analyze the impact of SGLT-2i/GLP-1RA on the relapses of CVD, which warrants further investigation through additional research. Finally, the population in this study was from only one municipal district in China, whereas a previous study had shown that the legacy effect of early glycemic control may be influenced by patient characteristics^7^, thus caution should be taken when extrapolating the findings of this study to populations with different characteristics.

In summary, among Chinese patients with newly-diagnosed T2D and without CVD at baseline, a significant association between mean HbA1c levels >7% during the early exposure period of 0–1, 0–2 years after diagnosis and a higher risk of subsequent CVD compared with HbA1c ≤ 7% was observed. Compared with non-users of SGLT-2i, GLP-1RA and DPP-4i, this association was smaller and non-significant when patients receiving SGLT-2i/GLP-1RA during the early exposure period. These findings may provide some evidence support for more extensive and earlier use of novel anti-hyperglycemic agents with cardiovascular protective effects such as SGLT-2i and GLP-1RA.

## Data availability statement

The datasets used and/or analyzed during the current study are available from the corresponding author SZ but restrictions apply to the availability of these data, which were used under license for the current study and therefore are not publicly available. Data are available from the authors upon reasonable request and with permission of the Yinzhou District Center for Disease Control and Prevention. Requests to access these datasets should be directed to SZ, siyan-zhan@bjmu.edu.cn.

## Ethics statement

The studies involving humans were approved by the ethical review committee of the Peking University Health Science Center (IRB. No: IRB00001052-18013-Exempt). The studies were conducted in accordance with the local legislation and institutional requirements. Written informed consent for participation was not required from the participants or the participants’ legal guardians/next of kin in accordance with the national legislation and institutional requirements.

## Author contributions

SD: Conceptualization, Data curation, Formal Analysis, Methodology, Validation, Writing – original draft, Writing – review & editing. HZ: Conceptualization, Methodology, Validation, Writing – original draft, Writing – review & editing. SC: Conceptualization, Writing – review & editing. YS: Resources, Software, Writing – review & editing. PS: Resources, Supervision, Writing – review & editing. HL: Resources, Supervision, Writing – review & editing. SZ: Conceptualization, Funding acquisition, Project administration, Supervision, Writing – review & editing.
