# Supplementary material for: Influence of early use of sodium-glucose transport protein 2 inhibitors, glucagon-like peptide-1 receptor agonists and dipeptidyl peptidase-4 inhibitors on the legacy effect of hyperglycemia
Source: Front Endocrinol (Lausanne). 2024 May 13;15:1369908. doi: 10.3389/fendo.2024.1369908 (PMC11128627; doi:10.3389/fendo.2024.1369908)
Supplement: Supplementary file 1 [file DataSheet_1.pdf]

Influence of early use of SGLT-2 inhibitors, GLP-1 and DPP4 inhibitors on the legacy effect of hyperglycemia

Siwei Deng[1,2,3], Houyu Zhao[1,4], Sanbo Chai[5], Yexiang Sun[6], Peng Shen[6], Hongbo Lin[6], Siyan Zhan*[1,2,3,7]

[1]Department of Epidemiology and Biostatistics, School of Public Health, Peking University, Beijing 100191, China.
[2]Center for Intelligent Public Health, Institute for Artificial Intelligence, Peking University, Beijing 100871, China.
[3]Key Laboratory of Epidemiology of Major Diseases (Peking University), Ministry of Education, Beijing 100191, China.
[4]School of Medicine, Chongqing University, Chongqing, 400030, China.
[5]Department of Endocrinology, Peking University International Hospital, Beijing 102206, China;
[6]Yinzhou District Center for Disease Control and Prevention, Ningbo 315100, China.
[7]Research Center of Clinical Epidemiology, Peking University Third Hospital, Beijing 100191, China.

Table of Contents

Table S1. Sodium-glucose cotransporter-2 inhibitors (SGLT-2i), glucagon-like peptide-1 receptor agonists (GLP-1RA), and dipeptidyl peptidase-4 inhibitors (DPP-4i) used in the study population……………………………………………………………………2

Table S2. Comorbidities used to define Charlson comorbidity index (CCI)…………3

Table S3. Characteristics of the study population by treatment groups during the early exposure period in two study cohort……………………………………………………4

Table S4. The incidence of outcomes in different treatment groups by the mean HbA1c levels during the early exposure period in two study cohort…………………………..6

Table S5. Characteristics between the participants with missing data and those without in two study cohort…………………………………………………………………..7

Table S1. Sodium-glucose cotransporter-2 inhibitors (SGLT-2i), glucagon-like peptide-1 receptor agonists (GLP-1RA), and dipeptidyl peptidase-4 inhibitors (DPP-4i) used in the study population

Drug class	ATC code	Drug name
SGLT-2i	A10BK01	dapagliflozin
	A10BK02	canagliflozin
	A10BK03	empagliflozin
	A10BK04	ertugliflozin
GLP-1RA	A10BJ01	exenatide
	A10BJ02	liraglutide
	A10BJ03	lixisenatide
	A10BJ04	albiglutide
	A10BJ05	dulaglutide
	A10BJ06	semaglutide
	A10BJ07	beinaglutide
	Not available	pegloxenatide
DPP-4i	A10BH01	sitagliptin
	A10BH02	vildagliptin
	A10BH03	saxagliptin
	A10BH04	alogliptin
	A10BH05	linagliptin
	A10BH06	gemigliptin
	Not available	trelagliptin

Table S2. Comorbidities used to define Charlson comorbidity index (CCI)

Comorbidities	Defined according to ICD-10 code	Adjustment in the model
Dementia	F00-F03, G30, G31.1, G31.8, G31.9	For calculating CCI, not included in the final analysis models
Chronic pulmonary disease	I27.8, I27.9, J40-J47, J60-J67, J68.4, J70.1, J70.3	Ditto
Connective tissue disease	M05, M06, M31.5, M32, M33, M34, M35.1, M35.3, M36.0	Ditto
Ulcer disease	K25-K28	Ditto
Mild liver disease	B18, K70.0-K70.3, K70.9, K71.3-K71.5, K71.7, K73, K74, K76.0, K76.2-K76.4, K76.8, K76.9, Z94.4	Ditto
Diabetic complication	E10.2-E10.5, E10.7, E11.2-E11.5, E11.7, E12.2-E12.5, E12.7, E13.2-E13.5, E13.7, E14.2-E14.5, E14.7	Ditto
Hemiplegia	G04.1, G11.4, G80.1, G80.2, G81, G82, G83.0, G83.1-G83.4, G83.9	Ditto
Moderate or severe renal disease	I12.0, I13.1, N03.2-N03.7, N05.2-N05.7, N18, N19, N25.0, Z49.0-Z49.2, Z94.0, Z99.2	Ditto
Any tumor (including lymphoma and leukemia except for malignant neoplasm of skin)	C00-C26, C30-C34, C37-C41, C43, C45-C58, C60-C76, C81-C85, C88, C90-C97	Ditto
Moderate or severe liver disease	I85.0, I85.9, I86.4, I98.2, K70.4, K71.1, K72.1, K72.9, K76.5-K76.7	Ditto
Metastatic solid tumor	C77-C80	Ditto
HIV/AIDS	B20-B22, B24, Z21	Ditto

Table S3. Characteristics of the study population by treatment groups during the early exposure period in two study cohort.

	Early exposure period: 1 year after T2D diagnosis				Early exposure period: 2 years after T2D diagnosis			
	SGLT2	DPP4	No user	*p*-value	SGLT2	DPP4	No user	*p*-value
No. of patients	1291	2648	17538		1129	3019	18345	
Age	50.09 ± 12.30	52.86 ± 12.19	54.02 ± 13.11	<0.0001	50.71 ± 12.42	52.80 ± 12.03	54.36 ± 12.56	<0.0001
Gender (% males)	880 (68.16)	1667 (62.95)	9983 (56.92)	<0.0001	761 (67.40)	1893 (62.70)	10366 (56.51)	<0.0001
Follow-up (mean ± SD, years)	1.20 ± 1.08	2.35 ± 1.66	3.90 ± 3.06	<0.0001	1.18 ± 1.24	2.19 ± 1.66	3.94 ± 2.98	<0.0001
HbA1c mean in exposure period ≤7.0%	604 (46.79)	1096 (41.39)	9256 (52.78)	<0.0001	526 (46.59)	1381 (45.74)	9960 (54.29)	<0.0001
Smoking	224 (17.35)	593 (22.39)	3860 (22.01)	<0.0001	235 (20.81)	705 (23.35)	4447 (24.24)	<0.0001
Smoking, NA	448 (34.70)	692 (26.13)	3890 (22.18)		304 (26.93)	676 (22.39)	3353 (18.28)	
Drinking	371 (28.74)	791 (29.87)	5725 (32.64)	<0.0001	366 (32.42)	991 (32.83)	6491 (35.38)	<0.0001
Drinking, NA	448 (34.70)	684 (25.83)	3876 (22.10)		304 (26.93)	672 (22.26)	3340 (18.21)	
**Education**								
University	166 (12.86)	255 (9.63)	1470 (8.38)	<0.0001	156 (13.82)	296 (9.80)	1376 (7.50)	<0.0001
High school	159 (12.32)	348 (13.14)	1745 (9.95)		157 (13.91)	406 (13.45)	1793 (9.77)	
Middle school	315 (24.40)	715 (27.00)	4551 (25.95)		262 (23.21)	861 (28.52)	4977 (27.13)	
Primary school	148 (11.46)	439 (16.58)	3832 (21.85)		148 (13.11)	507 (16.79)	4421 (24.10)	
Illiteracy	23 (1.78)	86 (3.25)	901 (5.14)		29 (2.57)	88 (2.91)	1078 (5.88)	
Other	168 (13.01)	350 (13.22)	2234 (12.74)		162 (14.35)	400 (13.25)	2368 (12.91)	
NA	312 (24.17)	455 (17.18)	2805 (15.99)		215 (19.04)	461 (15.27)	2332 (12.71)	
**CCI**								
0	382 (29.59)	921 (34.78)	7342 (41.86)	<0.0001	313 (27.72)	953 (31.57)	7015 (38.24)	<0.0001
1	367 (28.43)	677 (25.57)	4630 (26.40)		303 (26.84)	801 (26.53)	5028 (27.41)	
=2	260 (20.14)	503 (19.00)	2922 (16.66)		204 (18.07)	570 (18.88)	3238 (17.65)	
>2	282 (21.84)	547 (20.66)	2644 (15.08)		309 (27.37)	695 (23.02)	3064 (16.70)	
ACEI	142 (11.00)	221 (8.35)	1789 (10.20)	0.006	150 (13.29)	315 (10.43)	2210 (12.05)	0.0133

ARB	602 (46.63)	1008 (38.07)	6605 (37.66)	<0.0001	560 (49.60)	1254 (41.54)	7916 (43.15)	<0.0001
Statins	680 (52.67)	1270 (47.96)	6456 (36.81)	<0.0001	643 (56.95)	1517 (50.25)	7363 (40.14)	<0.0001
CCB	451 (34.93)	875 (33.04)	6465 (36.86)	0.0004	446 (39.50)	1122 (37.16)	7689 (41.91)	<0.0001
Beta-blocker	269 (20.84)	471 (17.79)	3191 (18.19)	0.0462	273 (24.18)	611 (20.24)	3681 (20.07)	0.0038
Diuretic	156 (12.08)	286 (10.80)	2545 (14.51)	<0.0001	152 (13.46)	382 (12.65)	3091 (16.85)	<0.0001
Other antihypertensive agents	66 (5.11)	138 (5.21)	915 (5.22)	0.9867	75 (6.64)	164 (5.43)	973 (5.30)	0.1530
Other lipid lowering agents	343 (26.57)	529 (19.98)	2653 (15.13)	<0.0001	313 (27.72)	657 (21.76)	3028 (16.51)	<0.0001
Metformin	828 (64.14)	1787 (67.48)	7152 (40.78)	<0.0001	812 (71.92)	2160 (71.55)	8747 (47.68)	<0.0001
Sulfonylureas	296 (22.93)	860 (32.48)	5417 (30.89)	<0.0001	351 (31.09)	1220 (40.41)	7235 (39.44)	<0.0001
Insulin	288 (22.31)	642 (24.24)	2488 (14.19)	<0.0001	264 (23.38)	754 (24.98)	2898 (15.80)	<0.0001
Alpha-glucosidase inhibitors	353 (27.34)	905 (34.18)	4283 (24.42)	<0.0001	375 (33.22)	1173 (38.85)	5241 (28.57)	<0.0001
Glinides	54 (4.18)	206 (7.78)	1298 (7.40)	<0.0001	64 (5.67)	310 (10.27)	1831 (9.98)	<0.0001
Thiazolidinediones	54 (4.18)	145 (5.48)	1055 (6.02)	0.0177	71 (6.29)	222 (7.35)	1535 (8.37)	0.0114

Table S4. The incidence of outcomes in different treatment groups by the mean HbA1c levels during the early exposure period in two study cohort

	Early exposure period: 1 year after T2D diagnosis				Early exposure period: 2 years after T2D diagnosis			
	mean HbA1c levels≤7.0%		mean HbA1c levels>7.0%		mean HbA1c levels≤7.0%		mean HbA1c levels>7.0%	
	Cases/Person years	Incidence (/100 000PY)	Cases/Person years	Incidence (/100 000PY)	Cases/Person years	Incidence (/100 000PY)	Cases/Person years	Incidence (/100 000PY)
**Primary analysis**								
Overall	868/14108061	6.2	934/13674666	6.8	1043/15364303	6.8	1047/13908234	7.5
SGLT-2i/GLP-1RA users	21/257761	8.1	25/308169	8.1	21/225754	9.3	25/258757	9.7
DPP-4i users	39/937922	4.2	79/1328568	5.9	65/1106937	5.9	99/1308459	7.6
Non-users	808/12912378	6.3	830/12037929	6.9	957/14028612	6.8	923/12341018	7.5
**Age group**								
**<55 years old**								
Overall	216/7610626	2.8	286/7719502	3.7	274/8116787	3.4	321/7702394	4.2
SGLT-2i/GLP-1RA users	7/160560	4.4	7/201946	3.5	8/139429	5.7	6/160037	3.7
DPP-4i users	9/567969	1.6	36/813032	4.4	18/636460	2.8	39/802457	4.9
Non-users	200/6882097	2.9	243/6704524	3.6	248/7340898	3.4	276/6739900	4.1
**≥55 years old**								
Overall	652/6497435	10.0	648/5955164	10.9	769/7244516	10.6	726/6205840	11.7
SGLT-2i/GLP-1RA users	14/97201	14.4	18/106223	16.9	13/86325	15.1	19/98720	19.2
DPP-4i users	30/369953	8.1	43/515536	8.3	47/470477	10.0	60/506002	11.9
Non-users	608/6030281	10.1	587/5333405	11.0	709/6687714	10.6	647/560118	11.6

Table S5. Characteristics between the participants with missing data and those without in two study cohort.

Variables	Early exposure period: 1 year after T2D diagnosis			Early exposure period: 2 years after T2D diagnosis		
	Without missing data	With missing data	*p*-value	Without missing data	With missing data	*p*-value
No. of patients	16446	5031		18159	4334	
Age	53.80 ± 12.86	53.14 ± 13.38	0.0015	54.15 ± 12.38	53.20 ± 13.05	<0.0001
Sex (% males)	9571 (58.20)	2959 (58.82)	0.4359	10470 (57.66)	2550 (58.84)	0.1576
Follow-up (mean ± SD, years)	3.90 ± 3.07	2.39 ± 2.13	<0.0001	3.86 ± 2.98	2.32 ± 2.05	<0.0001
**CCI**						
0	6483 (39.42)	2162 (42.97)	<0.0001	6557 (36.11)	1724 (39.78)	<0.0001
1	4498 (27.35)	1176 (23.38)		5125 (28.22)	1007 (23.23)	
=2	2801 (17.03)	884 (17.57)		3242 (17.85)	770 (17.77)	
>2	2664 (16.20)	809 (16.08)		3235 (17.81)	833 (20.48)	
ACEI	1833 (11.15)	319 (6.34)	<0.0001	2346 (12.92)	329 (7.59)	<0.0001
ARB	6600 (40.13)	1615 (32.10)	<0.0001	8190 (45.10)	1540 (35.53)	<0.0001
Statins	6573 (39.97)	1833 (36.43)	<0.0001	7835 (43.15)	1688 (38.95)	<0.0001
CCB	6315 (38.40)	1476 (29.34)	<0.0001	7831 (43.12)	1426 (32.90)	<0.0001
Beta-blocker	3127 (19.01)	804 (15.98)	<0.0001	3793 (20.89)	772 (17.81)	<0.0001
Diuretic	2482 (15.09)	505 (10.04)	<0.0001	3120 (17.18)	505 (11.65)	<0.0001
Other antihypertensive agents	823 (5.00)	296 (5.88)	0.0141	938 (5.17)	274 (6.32)	0.0024
Other lipid lowering agents	2802 (17.04)	723 (14.37)	<0.0001	3321 (18.29)	677 (15.62)	<0.0001
SGLT-2i/ GLP-1RA	843 (5.13)	448 (8.90)	<0.0001	825 (4.54)	304 (7.01)	<0.0001
DPP-4i	1956 (11.89)	692 (13.75)	0.0004	2343 (12.90)	676 (15.60)	<0.0001
Metformin	7637 (46.44)	2130 (42.34)	<0.0001	9636 (53.06)	2083 (48.06)	<0.0001
Sulfonylureas	5381 (32.72)	1192 (23.69)	<0.0001	7482 (41.20)	1324 (30.55)	<0.0001
Insulin	2396 (14.57)	1022 (20.31)	<0.0001	2942 (16.20)	974 (22.47)	<0.0001
Alpha-glucosidase inhibitors	4233 (25.74)	1308 (26.00)	0.7122	5438 (29.95)	1351 (31.17)	0.1143

Glinides	1222 (7.43)	336 (6.68)	0.0720	1777 (9.79)	428 (9.88)	0.8585
Thiazolidinediones	1036 (6.30)	218 (4.33)	<0.0001	1590 (8.76)	238 (5.49)	<0.0001

* Abbreviations: T2D, type 2 diabetes; SD, standard deviations; NA, not available; CCI, Charlson comorbidity index; ACEI, angiotensin converting enzyme inhibitors; ARB, angiotensin receptor blocker; CCB, calcium channel blocker; SGLT-2i, sodium-glucose transport protein 2 inhibitors; GLP-1RA, glucagon-like peptide-1 receptor agonists; DPP-4i, dipeptidyl peptidase-4 inhibitors.
